# Oral Intake of Carboxymethyl-Glucan (CM-G) from Yeast (*Saccharomyces uvarum*) Reduces Malondialdehyde Levels in Healthy Men

**DOI:** 10.3390/molecules200814950

**Published:** 2015-08-14

**Authors:** Vilma Barbosa da Silva Araújo, Adma Nadja Ferreira de Melo, Neyrijane Targino de Souza, Vânia Maria Barboza da Silva, Raul H. Castro-Gomez, Alexandre Sérgio Silva, Evandro Leite de Souza, Marciane Magnani

**Affiliations:** 1 Laboratório de Bioquímica de Alimentos, Departamento de Engenharia de Alimentos, Universidade Federal da Paraíba, João Pessoa, Paraíba 58051-900, Brazil; E-Mails: vilmaengenheira2@gmail.com (V.B.S.A.); admafdmelo@gmail.com (A.N.F.M.); neyrijane_14@hotmail.com (N.T.S.); nutvania@gmail.com (V.M.B.S.); 2 Departamento de Ciência e Tecnologia de Alimentos, Universidade Estadual de Londrina, Paraná 8606970, Brazil; E-Mail: rcastrog@yahoo.com; 3 Departamento de Educação, Universidade Federal da Paraíba, João Pessoa, Paraíba 58051-900, Brazil; E-Mail: ass974@yahoo.com.br; 4 Laboratório de Microbiologia de Alimentos, Departmento de Nutrição, Universidade Federal da Paraíba, João Pessoa, Paraíba 58051-900, Brazil; E-Mail: evandroleitesouza@pq.cnpq.br

**Keywords:** carboxymethyl-glucan, glucan-derivatives, malondialdehyde, blood cells

## Abstract

Carboxymethyl-glucan (CM-G) is a water-soluble derivative of β(1→3)(1→6) glucan, a well-known immunostimulant and antioxidant compound. In this experimental, randomized and placebo-controlled study, the effects of oral CM-G intake over a 60-day period on the peripheral blood, cholesterol, glycemic index and malondialdehyde (MDA) levels of healthy men was assessed. The CM-G was obtained from spent brewer’s yeast (*S. uvarum*) with DS 0.8 and molecular weight of 2.2 × 10^5^ Da. Following CM-G administration, no changes were observed in red and white blood cell, hematocrit, hemoglobin and platelet counts, or in cholesterol and glycemic indices. After 30 days of CM-G administration, the MDA levels decreased significantly (*p* ≤ 0.05) in men receiving CM-G. The results showed for the first time that CM-G may act as an adjuvant in preventing oxidative damage in healthy humans.

## 1. Introduction

β-d-glucan from the *Saccharomyces* cell wall is a polymer composed of repeating glucose units organized in a central skeleton linked by β (1→3) glycosidic bonds with side chains of varying size that are joined by β (1→6) linkages [1]. In the last decades, this polymer has been studied due its biological properties, particularly its immunomodulation activity [2,3]. Some authors have stated that soluble derivatives of (1→3)(1→6)-β-d-glucan present advantages such as higher activity and absence of toxicity and adverse effects when administered [3,4], however comparative studies showed the same effects for the both versions. Specially, an important factor of the use of (1→3)(1→6)-β-d-glucan soluble derivatives is that in this form, these substances can cross the gastrointestinal wall without causing damage to the digestive system, even when orally administered [5,6].

Among the soluble derivatives obtained from the (1→3)(1→6)-β-d-glucan of *Saccharomyces* spp., carboxymethyl-glucan (CM-G) is one of the most studied. CM-G is of particular interest to researchers due to its bioactive properties when ingested as a food supplement and because of its long safety record [3,4,7]. This polymer has significant bioprotective properties, such as antimutagenic, antigenotoxic, antioxidant and anticancer effects [4,8,9,10]. The main proposed mechanism behind these protective effects is the capability to scavenge reactive oxygen species (ROS) at low concentrations [11], with activity compared to α-tocopherol [12]. Due to its immunomodulatory and antioxidant effects, yeast glucan, particularly the CM-G, has been studied in clinical trials as a component of therapy for a variety of diseases, including prostate cancer [3,7,13,14]. However, because the majority of these studies involved patients with cancer or hyperlipidemia, information regarding the effects or benefits of CM-G intake for healthy individuals is still scarce. 

Lipid peroxidation (LPO) mediated by ROS has been implicated in many diseases. Malondialdehyde (MDA), a product of LPO, has been adopted as a measure of free radical production and therefore an “index of LPO” [15,16]. Studies involving experimental models of induced oxidative damage demonstrated that local or systemic administration of yeast β-glucan, through its antioxidant activity, could decrease MDA levels [11,16,17]. The level of MDA indicates the degree of oxidative stress, thus substances or compounds capable to decrease MDA in plasma or organs are beneficial and could be considered as therapeutic agents because attenuate the oxidative injuries in the body [15,18].

As some researchers have reported that lifestyle changes lead to increased consumption of food and compounds thought to be associated with health, especially compounds that reduce oxidative damage in the body [19,20], this study assessed the effects of the oral CM-G intake on the peripheral cells, cholesterol, MDA and blood glucose levels of healthy individuals. 

## 2. Results and Discussion

No changes were observed in the kidney and liver function of the men receiving CM-G as assessed by some well-known function-indicator enzymes and/or proteins (Table 1), and no side effects associated with CM-G were recorded, reinforcing early findings regarding the safety of CM-G [3,4,7].

**Table 1:** Results of liver and kidney function tests in healthy men before and after intake of CM-G for 60 days.

Liver Function	before CMG	after CMG	Reference Value (Method)
Transaminase Aspartate amino transferase (TGO)	25.1 ± 1	24.9 ± 0.9	11–41 U/L (automated kinetics)
Transaminase Alanine amino transferase (TGP)	10.2 ± 0.5	10.4 ± 0.8	7–52 U/L (automated kinetics)
Albumin	3.50 ± 0.75	34.30 ± 0.50	3.35–5.62 g/dL (capillary electrophoresis)
Direct bilirubin	0.07 ± 0.01	0.08 ± 0.02	up to 0.3 mg/dL (colorimetric)
Indirect bilirubin	0.33 ± 0.12	0.32 ± 0.13	up to 0.7 mg/dL (colorimetric)
Alkaline phosphatase	41.2 ± 10	40 ± 9	27–100 U/L (automated kinetics)
**Kidney Function**	**before CMG**	**after CMG**	**Reference Value (Method)**
Urea	38 ± 9	37 ± 10	10–52 mg/dL (automated enzymatic)
Creatinine	0.85 ± 0.23	0.89 ± 0.23	1.30 mg/dL (automated kinetics)

**Figure 1:**
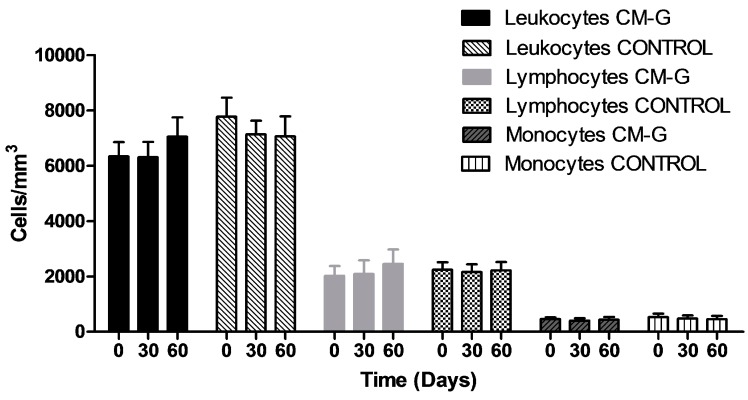
Average total leukocyte, monocytes and typical lymphocytes counts in healthy men before and after intake of carboxymethyl-glucan (CM-G). Bars on the columns show the standard deviation.

After CM-G intake, the total leukocyte counts did not increase significantly (*p* > 0.05). Additionally, no increase for the typical lymphocytes, monocytes and neutrophils were observed (Figure 1). 

These results are in accordance with the findings reported by Demir *et al*. [14], who observed no changes in leukocyte counts after 14 days of oral β-glucan administration to women undergoing cancer treatment. In contrast, Magnani *et al*. [3] observed a significant increase in total leukocyte counts, with an increase in typical lymphocytes, monocytes and neutrophils in men with advanced prostate cancer treated with CM-G by a shorter period than that studied here. Some models have demonstrated the ability of β-glucans and its derivatives administered by different routes to raise blood cell counts after leukopenia secondary to cancer treatments [4,9,21,22,23]. However, one hypothesis is that increases in blood cell counts in cancer patients receiving β-glucans are in part a physiological response to internal signals, such as low cell counts or inflammation. A previous study noted that the increase in blood cells was more pronounced in cancer patients with leukopenia when compared to those with normal counts before receiving CM-G [3]. 

No significant differences (*p* ≤ 0.05) were found in total (168.7 ± 16.9 before; 167.8 ± 16.9 after 60 days) HDL- (168.7 ± 14.5 before; 167.8 ± 14.9 after 60 days) or LDL- (37.4 ± 14.9 before; 386.8 ± 14.1 after 60 days) cholesterol levels after 60 days of CM-G intake. β-Glucans from yeast possess anti-hyperlipidemic properties, and consumption of β-glucans has been related to reductions in total cholesterol and LDL-cholesterol levels [24]. However, these effects are described for insoluble forms of glucans, which act as dietary fiber. In our study, two aspects could explain our finding of no changes in cholesterol levels: first, low amounts of CM-G were administered; second, the soluble form was used, which is able to pass from the gastrointestinal tract into systemic circulation. Orally administered soluble derivatives of β-glucan are absorbed through the gastrointestinal wall and pass into the circulatory system [5], activating immune pathways such as Dectin-1, CR-3, SIGNR1, TLR-2/6 and 4 [25]. 

**Figure 2:**
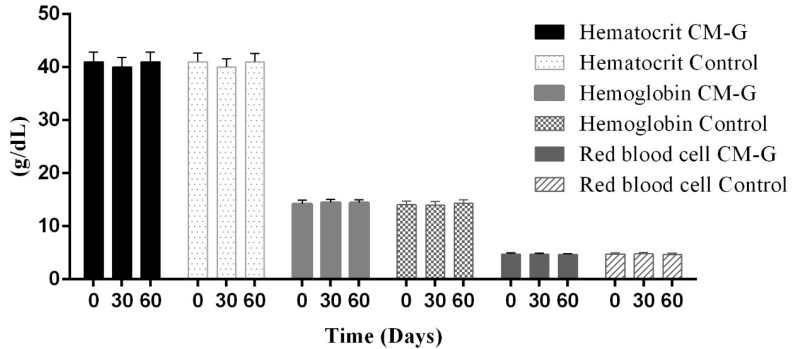
Average hematological data (hematocrit, hemoglobin, red blood cells) in healthy men before and after intake of carboxymethyl-glucan (CM-G). Bars on the columns show the standard deviation.

After 30 or 60 days of CM-G intake, no significant increase (*p* > 0.05) in red blood cells, hematocrit, hemoglobin or platelet counts was observed (Figure 2). Although some studies report that CM-G acts directly on myeloid progenitors, contributing to hematopoietic regeneration [26,27], early studies have had interesting results regarding changes in platelets and other hematimetric indices following oral administration of β-glucan derivatives in cancer patients. Increases in hemoglobin and platelet levels in cancer patients following 28 days of oral β-glucan administration have been reported [21]. 

**Figure 3:**
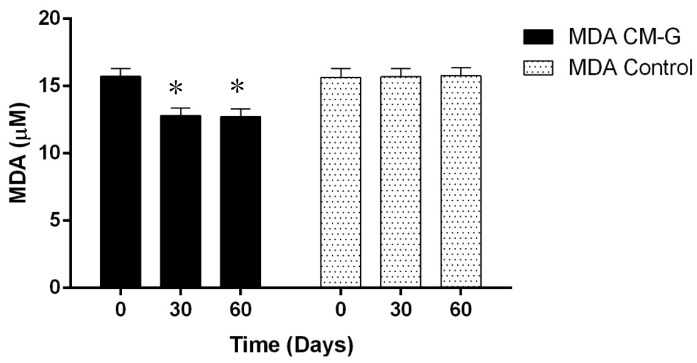
Average malondialdehyde (MDA) levels in healthy men before and after intake of carboxymethyl-glucan (CM-G). Bars on the columns show the standard deviation; * denotes significant differences (*p* ≤ 0.05) in MDA levels of individuals receiving CMG in comparison with baseline values.

Similarly, Magnani *et al*. [3] observed an increase in erythrocyte, platelet, hematocrit and hemoglobin levels after 28 days of CM-G administration in men with advanced prostate cancer, especially in patients initially presenting values below the minimum reference value. This further supports the hypothesis that β-glucan’s action is triggered by signals in the body, considering that in its soluble form acts synergistically with *in vivo* myeloid growth factors and cell signaling, improving hematopoietic recovery and mobilizing progenitor cells in the peripheral blood [3,28,29]. The MDA levels of the group receiving CM-G were reduced (*p* ≤ 0.05) after 30 and 60 days of intake when compared to the placebo group, which had no changes in MDA levels (Figure 3). This likely occurred because the ability of the CM-G to scavenge free-radicals [4,12], reducing the oxidative process and consequently the formation of LPO products (e.g., MDA). In a previous study with chronic uremic patients, a reduction of MDA plasma levels and increase of superoxide dismutase (SOD-1) activity during the administration of the commercial yeast β-glucan Zymosan^®^ was observed, however the particular condition of those individuals make difficult a deep comparison of these findings with the results obtained in our study [17]. Decreased MDA levels associated with the administration of soluble-yeast glucans were also reported in models of burn-induced oxidative organ damage in rats [15] and induced gastric damage [16]. Considering that MDA is an important measure of LPO, which is associated with oxidative damage (particularly in membranes), intake of CM-G could be an alternative to prevent oxidation, which naturally occurs in cells under the influence of many factors, including aging [30].

## 3. Experimental Section

### 3.1. Individuals and Experimental Design

The study was described as experimental, randomized and placebo-controlled and was conducted after the approval of the Committee on Ethical Research Involving Humans Beings of the Federal University of Paraíba (João Pessoa, Brazil), under the Process Number 10734712.8.0000.5188. The individuals were selected from the Civil Police Staff of João Pessoa city, Paraíba State, Brazil. Only men were included in the study to exclude any menstrual cycle influence. Menstruation and ovulation are the main events during the menstrual cycle [31]. A total of 52 healthy men ranging between 26 and 37 years of age (median age 29 ± 2.68 years) were distributed in two groups of 26 individuals (placebo and CM-G-administrated) after giving their written informed consent. Exclusion criteria included the regular consumption of alcohol, tobacco smoke and/or the use of medications for chronic or congenital diseases. The inclusion criteria included an age greater than 22 years, normal blood cell levels, normal blood glucose and cholesterol levels, participation in physical activity at least two times a week and consumption of a diet including salad and fruit an average of three times a week. The trial was performed during the 60 days and the individuals were contacted weekly to monitor their condition and to report any adverse effects associated with the trial. 

### 3.2. Extraction of CM-G

CM-G was obtained from the cell wall of *Saccharomyces uvarum* discarded from a brewery as slurry, with a substitution degree of 0.8, according to a procedure described elsewhere [3]. The brewer’s yeast slurry was sieved through a 0.297 mm mesh, and distilled water was added (30%, *w*/*v*). To remove residual ethanol, the suspension was washed three times with distilled water (5000× *g* for 5 min at 10 °C), NaCl was added (3%, *w*/*v*), and the suspension was autolyzed in a bath under stirring (120 rpm) at 55 °C for 24 h. The temperature was then increased to 85 °C and maintained for 5 min to inactivate the cellular enzymes. After the suspension reached room temperature, the yeast cell wall was obtained by centrifugation at 4500× *g* and 4 °C for 10 min. The insoluble material from the autolyzed brewer’s yeast slurry was diluted in sodium phosphate buffer (30% *w*/*v*; 0.02 M sodium phosphate buffer, pH 7.5), heated to 121 °C (1.5 atm) in an autoclave for 4 h and washed three times with distilled water (4500× *g* for 7 min at room temperature). To extract β-glucan, sonication (20 kHz; 150 W; 6 min), lipid extraction using petroleum ether (2 h under reflux) and proteolysis using the enzyme Protemax^®^N200 (5 h at 55 °C and pH 7.5; 0.4 U per gram of cell wall in a 20% aqueous suspension) were performed. After proteolysis, the insoluble residue (β-glucan) was washed five times with distilled water by centrifugation (4500× *g* for 5 min at room temperature). The derivatization of β-d-glucan to CM-G was performed using monochloro acetic acid and the substitution degree (DS) achieved was determined by potentiometric titration with potassium hydroxide solution. CM-G was ultrasonicated (1 g/100 mL distilled water) at 20 kHz and 100 W in an ice bath, dialyzed (48 h against distilled water under mild agitation with frequent water exchange), frozen at −20 °C and lyophilized.

For assays, CM-G or placebo (maltodextrin) was divided into 50 mg portions, packed in metalized BOPP bags and hermetically sealed. The dose of oral administration was based on earlier studies [3,4,7], as well as in results of preliminary studies. The chemical identity of the polymer was confirmed infrared analysis (IR). The IR spectrum was obtained on a model 3300 FT-IR spectrophotometer (Shimadzu, Tokyo, Japan). KBr pellets were used for preparation of the samples, and the deviation of the measurements was ±2 cm^–1^. 

### 3.3. Blood Samples and Analysis 

Peripheral venous blood samples were drawn for initial values from the individuals on day 1 of the study before the ingestion of CM-G with breakfast. Early each morning, individuals ingested a 50 mg CM-G or placebo with breakfast. After 30 and 60 days, blood samples were recollected and analyzed. Both samples were taken while patients were fasting. Four-and-a-half milliliter Vaccutainer™ tubes containing EDTA were used, and all the samples were processed immediately after collection by an automatic method, using an Abbott Cell Dyn 3200 instrument (San Diego, CA, USA) for blood cells counts. Blood glucose levels were determined by an automated spectrophotometric system (Baker Instruments, Allentown, PA, USA), and total LDL- and HDL-cholesterol levels (mg/dL) were calculated according to the method described by Nicolosi *et al.* [24]. Levels of transaminases, albumin, direct and indirect bilirubin and alkaline phosphatase, as well as levels of urea and creatinine in serum were assessed before and after intake of CM-G for 60 days to verify the liver and kidney function [3]. MDA was determined after antioxidant treatment of the plasma sample, followed by a protein precipitation step using trichloroacetic acid, acid hydrolysis and formation of a MDA thiobarbituric acid complex. The MDA-(TBA)2 adduct was separated from other interfering compounds by C18 reverse-phase HPLC techniques, with visible detection at 532 nm [32].

The results obtained before and after CM-G or placebo intake were analyzed using the Wilcoxon signed-rank test and the *t* test for dependent paired samples. A *p* value ≤0.05 indicated significant differences. Sigma Stat 3.5 computer software (Jandel Scientific Software, San Jose, CA, USA) was used for the statistical analysis of the data.

## 4. Conclusions

Our findings are worthy of note considering that CM-G was administered with food and to healthy individuals, suggesting that the antioxidant properties of CM-G derived from yeast are not related to disease-signaling in the body and are not compromised when ingested with foods. Therefore, these results suggest that CM-G may act as an adjuvant in preventing oxidative damage in humans. Besides, to the best of our knowledge, this is the first report of a decrease in blood MDA levels in healthy men following the intake of yeast CM-G with food.
